# Physicochemical Characteristics of Yogurt from Sheep Fed with *Moringa oleifera* Leaf Extracts

**DOI:** 10.3390/ani12010110

**Published:** 2022-01-04

**Authors:** Miriam M. Mendoza-Taco, Aldenamar Cruz-Hernández, Angélica A. Ochoa-Flores, Josafat A. Hernández-Becerra, Armando Gómez-Vázquez, Victor M. Moo-Huchin, Ángel Piñeiro-Vázquez, Alfonso J. Chay-Canul, Einar Vargas-Bello-Pérez

**Affiliations:** 1 División Académica de Ciencias Agropecuarias, Universidad Juárez Autónoma de Tabasco, Carretera Villahermosa-Teapa, km 25, R/A, la Huasteca 2ª Sección, Villahermosa 86280, Tabasco, Mexico; marlenymendozazootecnia1@gmail.com (M.M.M.-T.); ingaldecruz@gmail.com (A.C.-H.); angelica.ochoa@ujat.mx (A.A.O.-F.); dragv2@hotmail.com (A.G.-V.); 2 División de Tecnología de Alimentos, Universidad Tecnológica de Tabasco, Villahermosa 86288, Tabasco, Mexico; jahernandez.tc@uttab.edu.mx; 3 Tecnológico Nacional de México, Instituto Tecnológico de Mérida, km 5 Mérida-Progreso, Mérida 97118, Yucatán, Mexico; vmmoo@yahoo.com; 4 Tecnológico Nacional de México, Instituto Tecnológico de Conkal, Avenida Tecnológico s/n Conkal, Conkal 97345, Yucatán, Mexico; angel.pineiro@itconkal.edu.mx; 5 Department of Veterinary and Animal Sciences, Faculty of Health and Medical Sciences, University of Copenhagen, Grønnegårdsvej 3, DK-1870 Frederiksberg C, Denmark

**Keywords:** *Moringa oleifera* extracts, physicochemical composition, milk, yogurt

## Abstract

**Simple Summary:**

This study determined the effect of feeding *Moringa oleifera* (MO) leaf extracts to lactating ewes on the physicochemical composition of their milk and yogurt during storage (4 °C for 14 days) and the sensory acceptance of the yogurt. The supplementation of MO in lactating ewes’ diets improved the contents of protein, ash, acidity, and leucine in their milk. The effect of MO in yogurt showed an increase in nonfat solids, protein, and leucine during storage at 4 °C for 14 days. The MO had a positive effect on the overall acceptance of yogurt at a higher MO level of supplementation in sheep diets. Therefore, adding MO leaf extracts to sheep diets could be a feasible nutritional strategy to improve the physicochemical characteristics of yogurt from sheep.

**Abstract:**

This study determined the effect of feeding *Moringa oleifera* (MO) leaf extracts to lactating ewes on the physicochemical composition of their milk and yogurt during storage (4 °C for 14 days) and the sensory acceptance of the yogurt. Over 45 days, 24 multiparous lactating Pelibuey and Katahdin ewes (two days in lactation) were randomly assigned to four groups: MO-0, basal diet (BD) + 0 mL MO; MO-20, BD + 20 mL MO; MO-40, BD + 40 mL MO; and MO-60, BD + 60 mL MO. In the milk, an increase of 6% in protein, 26% in leucine, 14% in ash, and 1% in the pH (6.71) was observed with MO-60. The density values decreased by 0.3% at a higher dose of MO compared to MO-0, while the nonfat solids (NFS) in the milk were similar between the treatments. In the yogurt, an increase of 5% in protein, 113% in leucine (MO-20), 9% in NFS, and a reduction of 2% in moisture with MO-60 was observed. The acidity reflected an inverse relationship to the pH, as did the moisture and NFS with MO-60. In conclusion, dietary supplementation with MO in lactating ewes did not have negative effects on the chemical composition of their yogurt during storage (14 days). Overall, feeding sheep with 20 mL of MO positively influenced the physicochemical composition of their milk and yogurt during storage.

## 1. Introduction

The importance of sheep’s milk yogurt lies in its amount of nutrients [1,2], such as peptides, fatty acids, and microelements [3]. In sheep’s milk, the oxidation process is naturally balanced by intrinsic antioxidants such as proteins and fats, especially casein, which is bound to specific amino acids [4]. The abundance of antioxidants in milk is dependent on the environmental and stressful conditions to which the animal may be exposed, causing an imbalance between oxidizing agents and antioxidants [1]. These changes in milk quality depend on the type of feed. Ruminants are mostly fed on forages; however, forages derived from shrubs contain high amounts of phenolic compounds, tannins, saponins, and flavonoids that are available at relatively low costs, making them a strategy for improving dairy product quality [5].

*Moringa oleifera* (MO) is a tropical shrub with forage potential, native to the Himalayas and distributed throughout the world, particularly in tropical and subtropical regions [6,7]. It is resistant to droughts, has a fresh material biomass yield of 25.8 to 41.18 tons per hectare per year, and is considered a phytogenic resource with a high potential for livestock [8]. Its foliage contains protein (>18% DM), fiber (32% DM), a low amount of tannins (12 g/kg DM), phytate (21 g/kg DM), the absence of trypsin and amylase inhibitors [9,10], 845 mg/kg of calcium, 108 mg/kg of phosphorus, and 421 mg/kg of potassium [8]. The use of MO in sheep diets has been shown to have effects on the productive parameters (higher DM intake, improved daily milk production, and increased weight gains) and nutritional quality of dairy products [5,11,12].

There are studies pointing to the fact that MO (2, 3, and 4 g of MO per 100 g of cream cheese) can improve nutritional content, flavor during storage, the growth of probiotic strains [13]. These results are in agreement with El-Gammal et al. [8], who added 0.4% MO to yogurt, resulting in improved nutritional values, sensory properties, and antimicrobial effect against *S. aureus*, *E. faecalis* and *B. cereus*, *E. coli*, and *S. Typhimurium*. Fardet and Rock [4] reported antioxidant activity as an important nutritional property in probiotic yogurts due to the presence of peptides released by probiotic proteolysis. Kholif et al. [2] mentioned that MO also modifies the concentration of unsaturated fatty acids. However, not much research focused on the effects of feeding ruminants with MO on dairy product quality is available.

High levels of catalase activity have also been reported in the milk of sheep that were fed MO, causing oxidative effects on the fats and the conservation of their nutritional characteristics [14], especially the quality of the lipids in dairy products [15]. In addition, yogurt made from sheep’s milk is a food with low atherogenic and thrombogenic risk, due to its lipid composition [16]. However, there is scarce information on MO extracts in animal feed as enhancers of the nutritional profile of milk and dairy products [17], which encourages further exploration of the effects of animal diets on dairy product quality [18]. Adding MO extract to sheep diets may improve the physicochemical characteristics of dairy products. In this study, yogurt was chosen as a food matrix that has not received much attention from the farm-to-fork approach. Thus, the objective of this study was to evaluate the physicochemical composition of yogurt made from sheep fed with *Moringa oleifera* leaf extract.

## 2. Materials and Methods

### 2.1. Animal Management and Experimental Design

The animals were handled according to the guidelines and regulations for animal experimentation of the Academic Division of Agricultural Sciences of the Universidad Juárez Autónoma de Tabasco (ID project PFI: UJAT-DACA-2015-IA-02). The study was carried out at “Rancho San Francisco”, located at 21°14′48″ N and 89°02′35″ L, at 5 m above sea level, in the municipality of Dzidzantun (Yucatán, Mexico). The average temperature was 26 °C, with 9.8 mm of rain during the experimental months (between November and December) and extremes of relative humidity between 66% and 89% [19,20]. Twenty-four clinically healthy sheep (Pelibuey and Katahdin) with 2 days of lactation were randomly assigned to four groups of six animals. Animals were 2 to 3 years of age, with a mean body weight (BW) of 35.7 ± 5.02 kg and a body condition score (BCS) of 2.07 ± 0.18 [21]. Groups were balanced for BW and no differences were found in the initial BW among feeding groups (*p* = 0.72). Sheep were housed in individual pens (2 × 3 m) and were managed under a feedlot system for 45 days with water ad libitum. The number of animals for each treatment was based on a similar study on dietary interventions [22].

At the beginning of the study, sheep were dewormed with Closantel 5%^®^ (Wyeth LLC, Madison, NJ, USA) at a dose of 10 mg/kg of body weight. The animals were fed a basal diet (offered at 08:00 h) based on ground corn, soybean meal, sugar cane molasses, minerals, vitamins, and stalks of fresh minced Taiwanese grass (*P. purpureum*, offered at 6:00 p.m.) as fodder, in a ratio of 80:20, respectively. The nutritional composition of the basal diet contained a metabolizable energy of 11.5 MJ/kg DM and 15% crude protein [23]. The basal diet was offered ad libitum, with feeding ratios designed to ensure a daily rejection margin of 10%. The diet was supplemented with a daily supply of MO at a dose of 0 mL (MO-0), 20 mL (MO-20), 40 mL (MO-40), and 60 mL (MO-60). These levels of MO per animal were used according to the levels of MO used by Kholif et al. [2] in goats. 

To avoid feed sorting, treatments were mixed manually with 200 g of concentrate; once the portion of feed was finished, the rest of the concentrate was provided. The diets were formulated to meet the theoretical requirements for dairy ewes with an average weight of 45 kg and an average milk yield of 1.74 kg/d, with 4.5% total protein and 4.5% fat, according to the Agricultural and Food Research Council guidelines [23].

### 2.2. Milk Samples

The ewes were manually milked twice a week during the mornings (07:00 h). The lambs of each ewe were removed 12 h before each milking (19:00 h). Before each milking, a dose of 3 IU of oxytocin was applied intramuscularly [24] to stimulate the secretion of the milk. The milk obtained was immediately cooled to 4 °C, and the corresponding daily individual production was recorded.

### 2.3. Moringa oleifera Extract

*Moringa oleifera* extract leaves were collected randomly from young and mature plants. The leaves were cut (1 to 2 cm long) and dried at 40 °C for 72 h in a forced-air oven and then crushed with a mill (0.5–1 mm). The extract was prepared starting from 1 g of MO leaf powder placed in an Erlenmeyer flask with 20 mL of water–ethanol solution (1:1 *v*/*v*). The extract was analyzed to determine the presence of bioactive compounds (hydrolyzed tannins, condensed tannins, phenolic compounds, saponins, and flavonoids). Details on extract preparation can be found in a companion paper [5]. 

### 2.4. Yogurt Manufacturing

The yogurt was processed at the experimental dairy plant of the Technological Institute of Mérida, following the protocol of Hekmat et al. [25] with some modifications, such as the incubation temperature of 37 °C to 45 °C and the percentage of added culture (4%). The milk collected was subjected to pasteurization at 85 °C for 30 min, and then it was allowed to cool to 45 °C for the inoculum of the microbial culture (Bioprox^®^-YP-700, France) composed of strains of *Lactobacillus bulgaricus* and *Streptococcus thermophilus*. Then, it incubated until reaching a drop in pH of 4.6. Subsequently, it was stored in a cold chamber at 6 ± 2 °C for subsequent physicochemical and sensory analysis.

### 2.5. Physicochemical Analysis of Milk and Natural Yogurt

Collected milk was pooled and four subsamples of 150 mL per treatment were taken for subsequent physicochemical analysis by the following methods described by the AOAC [26]. Titratable acidity (% lactic acid) was measured by titration with 0.1 N sodium hydroxide. The pH value was determined using a digital potentiometer (HI-2210, HANNA^®^, Ciudad de México, Mexico) by direct insertion of the electrode to the sample. Nonfat solids (NFS) were measured with a refractometer (ATAGO^®^; RX-5000, Tokyo, Japan) and nitrogen content by the Kjeldahl method 991.20 [26], milk protein was calculated as N × 6.38 and the percentage of ash per incineration as 945.46 [26], and the concentration of peptides was determined by the modified ninhydrin colorimetric method [27]. All analyses were carried out in triplicate. The experimental yogurt samples were stored in a cold chamber at 6 ± 2 °C and the parameters mentioned above were evaluated on days 1, 7, and 14 of storage.

### 2.6. Sensory Evaluation

The sensory evaluation of the yogurt was carried out by 70 consumers (40 women and 30 men aged between 20 and 50 years). Consumers were selected according to their availability for the study and their level of consumption of this type of product [28]. Subsequently, each consumer received four glasses with samples (10 mL) of each type of natural yogurt and a sensory evaluation sheet where each panelist evaluated the global appreciation of each sample using the 9-point hedonic scale (1 = I extremely dislike it and 9 = I like it extremely). Samples were randomly coded with three digits and analyzed as a sequential monadic test [29].

### 2.7. Experimental Design and Statistical Analysis

The chemical composition of the milk and natural yogurt and the sensory evaluation were analyzed using the SAS 9.4 statistical program. An analysis of variance was used to test the significance of the treatments with a completely randomized design and general linear model (GLM). For the chemical composition of natural yogurt, the data were analyzed with two study factors, considering the level of the extract of MO leaves (0, 20, 40, and 60 mL) and storage days (0, 7 and 14 days). Significant differences (*p* < 0.05) between means were determined by Tukey’s test with a significance of 5%. 

## 3. Results and Discussion

### Physicochemical Composition of Milk and Plain Yogurt

The dietary supplementation of extracts of *Moringa oleifera* leaves at doses of 20, 40, or 60 mL/d per ewe in lactating ewes did not affect the total milk yield (53 ± 5 kg) or the daily milk yield (1.17 ± 0.12 kg). In contrast to the content of nonfat solids and the milk density, the supplementation of MO in sheep feed resulted in an increase (*p* < 0.05) in the protein and ash contents at a higher dose of MO (Table 1). Except for the protein values, differences were observed in all variables (*p* < 0.05) of the physicochemical characteristics of the yogurt during storage at 4 °C for 14 days. The sheep that received 20 mL of MO showed a higher leucine content in their milk and yogurt compared to the other treatments.

**Figure 1:**
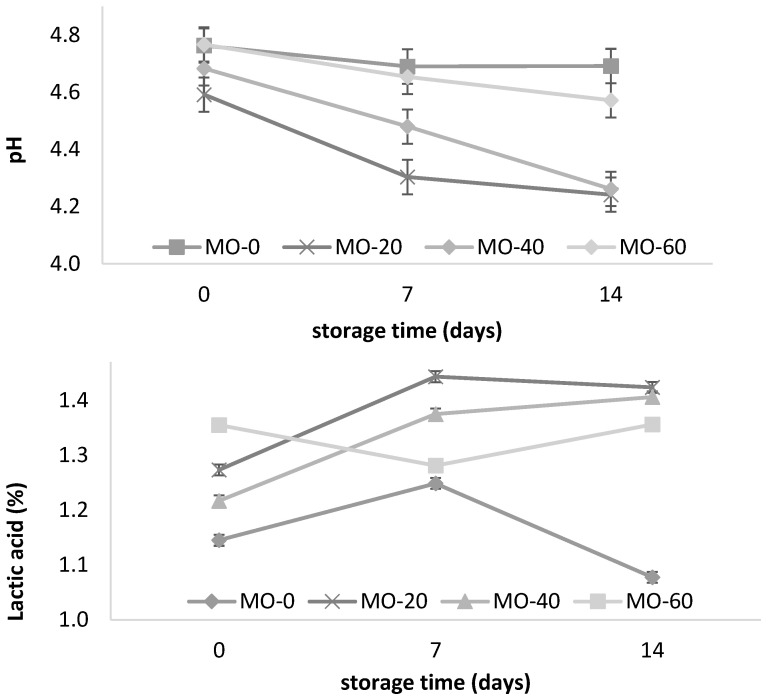
Storage changes in pH and acidity of yogurt from sheep fed with *Moringa oleifera* extracts. Bars denote standard error of the means.

The yogurt pH was decreased in the MO-20 and MO-40 groups at 14 days of storage (Table 2, Figure 1). The yogurt pH from the sheep fed 40 mL of MO gradually decreased from day one to day fourteen. A similar pattern was observed with the yogurt from the sheep that were fed 20 mL of MO. However, increasing the amount of MO to 60 mL in the diet did not promote pH changes in the yogurt, which resulted in a lower amount of lactic acid (Figure 1) at the end of storage. Similar values were reported by El-Gammal et al. [8] and Cardines et al. [30] in yogurts with the direct addition of MO leaf and seed extracts, where the results varied between 0.80 and 1.11 g of lactic acid/100 g of yogurt and a decrease in the pH from 4.65 to 4.11 for 15 to 28 days of storage. This tendency towards a decrease in the yogurt pH and an increase in the acidity during storage in the present study was consistent with the study of Güler and Gürsoy [31]. They attributed the high acidity values to the degradation of the lactose to lactic acid by the lactic acid bacteria; the higher content of acetaldehyde produced by the cleavage of threonine; the type of culture used in the fermentation, regardless of the type of milk; and the low content of diacetyl present in yogurt under refrigerated conditions. Paseephol et al. [32] and Balthazar et al. [16] attributed it to the continuous metabolism and enzymatic activity of crops during storage. With regard to the low acidity of the yogurts (Figure 1), this could be due to the influence of the high levels of total solids present in the milk [31].

However, pH changes in yogurts have also been attributed to the type of additive used during processessing, regardless of the type of feed that the sheep received. In this regard, Parra [33] points out that when adding 1% (p v-1) green tea to yogurt, the growth of lactic acid bacteria is stimulated, promoting pH values of 4.5 to 4.39 during storage (20 days) under refrigeration conditions (4 °C). In another study, Vazquez et al. [34] reported a decrease in the pH of fruity yogurts with mango and banana during storage (4.35 to 4.10 and 4.36 to 4.18, respectively), and an increase of 1.1/0.93% in the lactic acid. Balthazar et al. [16] reported an increase of 1–1.35% in the lactic acid content over 28 days of storage in yogurts containing inulin (2, 4, and 6%), and attributed this pattern to the persistent metabolic and enzymatic activity of lactic acid bacteria during low-temperature storage. The foregoing seems to affirm that the pH and acidity values in this study are within the ranges established by the Mexican standard NOM-181-SCFI-2010 and by the Codex Alimentarius Commission [35], which indicates a minimum acidity of 0.5–1.5% in cow’s milk. 

**Figure 2:**
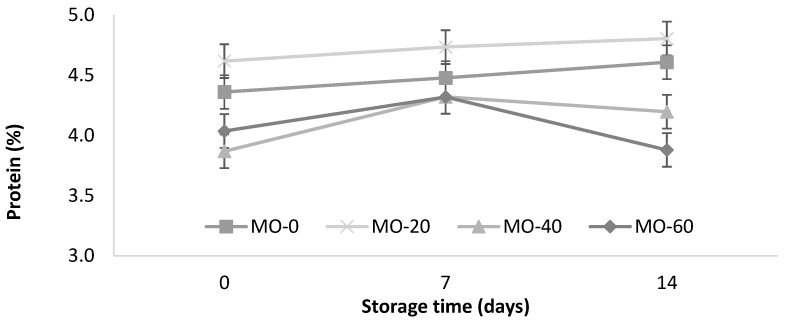

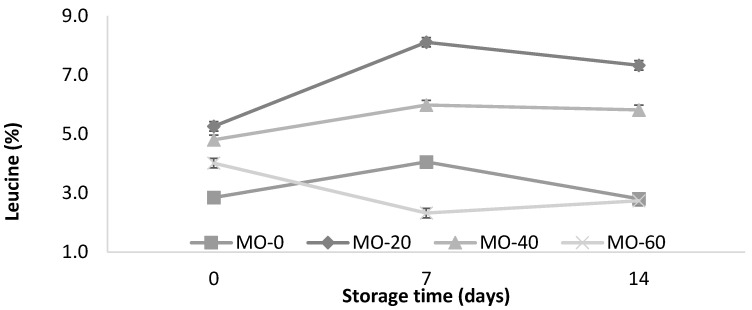
Storage changes in protein and leucine contents of yogurt from sheep fed with *Moringa oleifera* extracts. Bars denote standard error of the means.

As shown in Figure 2, a slight increase in the protein content (<0.0001) of the yogurt from the sheep that were fed with 40 mL of MO was observed. The protein increased with the storage time in the treatments MO-0 and MO-20 (4.36 to 4.61% and 4.62 to 4.80%, respectively). These values are within the minimum value (2.7%) of the Mexican standard for cow’s milk yogurt [35].

The protein values in this study were slightly lower than those reported by Güler and Gürsoy [31] in sheep’s milk yogurts (5.5 ± 0.65%) and higher than the values reported by Cardines et al. [30], who they reported values from 3.12 to 3.20 g/100 g in yogurts with the inclusion of MO seed extracts (0.5% and 1.5%) compared to 2.73 g/100 g in the control. In this study, the use of 20 mL of extract in the diet of the sheep significantly improved the protein levels compared to 40 and 60 mL of extract. On the other hand, the levels of the extracts did not promote protein modifications during storage. It is likely that the MO protein helped to improve the structural cohesion of the compounds, due to the greater binding capacity of water with the active sites of the protein added to the yogurt [30].

The casein micelles (the majority protein in milk, >80%) contained in dairy products (probiotic yogurts) have important properties such as polyphenol transport [3] and the potential antioxidant effect of their peptides (release by the proteolytic action of *Lactobacillus casei*/*acidophilus* bacteria), which participate in the reduction of oxidative stress [4]. This is why it is important to expand alternatives for food management systems that help improve the presence of milk bioactive compounds such as proteins (immunoglobulins, lactoferrin, and peptides), fats, minerals, oligosaccharides, and melatonin that are capable of providing protection against infections, immune activation, inflammation reduction, and antitumor and antimicrobial effects [3].

The results obtained regarding the protein content in the present study are consistent with those reported by Al-Juhaimi et al. [36] and Kholif et al. [17], who found slight increases in protein and ash in milk from goats supplemented with 25% leaves and 20 mL of MO extract, respectively. The positive effect of the increase in milk protein could be due to the result of greater ruminal fermentation and the high digestibility of protein and dry matter [17,37]. Kumar et al. [38] attributes the increase to phytobiotic compounds (bioactive compounds) that stimulated the secretion of digestive fluids, which would cause a positive change in ruminal fermentation. On the other hand, Babiker et al. [12] and Kekana et al. [39] reported that the protein content of milk was not affected by diets with MO, due to the adequate levels of fiber and protein in the animals’ diet. Sanchez et al. [40] mention that if a food contains the necessary amount of protein, the milk production from the animals that eat this food presents high levels of protein.

In addition, studies [41] have shown that the main contributor to the total antioxidant capacity (hydrophilic and lipophytic) of whole milk is the casein fraction, together with vitamin C and uric acid (hydrophilic). This should encourage us to obtain in subsequent studies higher antioxidant capacities in the milk of animals supplemented with extracts, due to the increase in protein levels recorded in this research.

**Figure 3:**
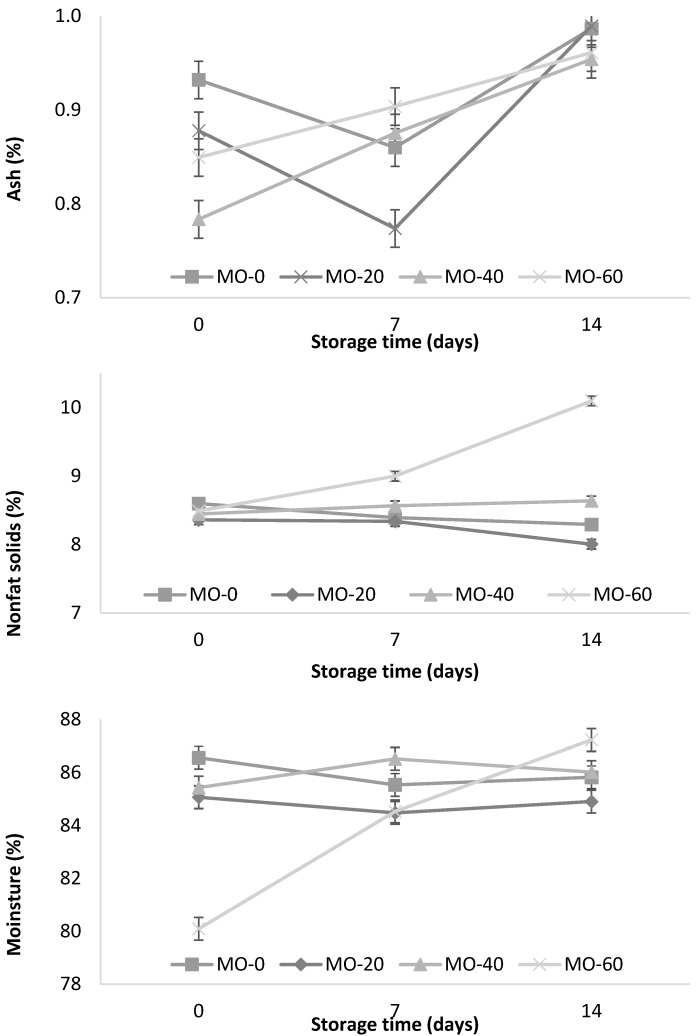
Storage changes in ash, nonfat solids, and moisture content of yogurt from sheep fed with *Moringa oleifera* extracts. Bars denote standard error of the means.

In Figure 3, the influence of MO on the ash content during yogurt storage is observed. The MO-40 and MO-60 treatments showed a significant increase (*p* < 0.001) during storage (0.79 to 0.96 and 0.85 to 0.96%, respectively) as well as the interactions between both factors (the level of MO inclusion and time of storage). On the other hand, the MO-0 and MO-20 treatments showed no significant differences. These results are consistent with those reported by Jung et al. [42], who mentioned that the ash content in the yogurt samples varied from 0.87 to 0.95% at a higher concentration of red ginseng extract (0.5, 1, 1.5, and 2%), and lower than those reported (1.29%) by Güler and Gürsoy [31].

In Figure 3, the temporal pattern of nonfat solids (NFS) during storage is observed. The NFS values of the yogurts were significantly (*p* < 0.001) higher in the animals supplemented with 60 mL of *M. oleifera* extract and during the 14 days of storage. Güler and Gürsoy [31] reported similar results (10.7%) when using cultures of bacterial strains *S. thermophilus* and *L. delbrueckii* subsp. *bulgaricus* (codes: CH-1 and YF-3331) during the fermentation of yogurt. On the other hand, Jung et al. [42] reported higher values (12.40%) in yogurts supplemented with red ginseng extracts (0.5, 1, 1.5, and 2%). This variation could be explained by the origin of the milk (breed, age, species, and animal feed) used in the yogurt fermentation. In Figure 3, the significant influence of MO on the moisture content during the storage period (14 days) is observed. The MO-60 treatment showed an increase during storage compared to the MO-0, MO-20, and MO-40 treatments.

The results of the sensory analysis revealed that feeding MO to lactating ewes did not have a significant effect on the overall acceptance of their yogurt. The overall acceptance of the yogurt varied from moderate to slightly dislike (Table 3). The MO-40 and MO-60 treatments obtained higher scores compared to MO-0. This may have been because the yogurt did not contain sugars. In this regard, Medina et al. [43] mentioned that the addition of sugars and sweeteners could positively influence consumer preference. In order to have more insight into the resulting yogurts, further studies should asses a full sensory analysis that accounts for parameters such as texture, odor, and flavor.

## 4. Conclusions

Feeding MO to lactating ewes improved the contents of protein, ash, acidity, and leucine in milk. The effect of MO on yogurt manifested as an increase in nonfat solids, protein, and leucine during storage at 4 °C for 14 days. Overall, feeding sheep with 20 mL of MO had the most favorable result for the physicochemical composition of milk and yogurt. 

## Figures and Tables

**Table 1:** Chemical and physical composition of milk from sheep fed with *Moringa oleifera* extracts.

Variables	*Moringa oleifera* (mL)					*p*-Value
	0	20	40	60	SE	
pH	6.64 ^ab^	6.62 ^b^	6.58 ^b^	6.71 ^a^	0.019	0.008
Acidity (%)	0.37 ^a^	0.34 ^b^	0.26 ^c^	0.35 ^b^	0.003	<0.001
Density (g/mL)	1.042 ^a^	1.039 ^c^	1.040 ^b^	1.039 ^c^	0.001	<0.001
Nonfat solids (%)	13.17	13.08	13.03	13.03	0.081	0.620
Leucine (mg/100 g protein)	2.72 ^b^	3.43 ^a^	2.63 ^b^	1.73 ^c^	0.066	<0.001
Protein (%)	4.26 ^ab^	4.34 ^ab^	4.22 ^b^	4.52 ^a^	0.072	0.037
Ash (%)	0.84 ^b^	0.96 ^a^	0.94 ^a^	0.96 ^a^	0.015	<0.001

^a,b,c^ Means in the same row with different superscripts differ significantly. SE: standard error.

**Table 2:** Chemical and physical composition of yogurt made from sheep fed with extracts of *Moringa oleifera* during storage.

Variables	*Moringa oleifera* (mL)					*p*-Value		
	0	20	40	60	SE	N	T	N×T
pH	4.72 ^a^	4.38 ^b^	4.48 ^b^	4.66 ^a^	0.033	<0.0001	<0.0001	0.049
Ash	0.93 ^a^	0.88 ^ab^	0.87 ^b^	0.90 ^ab^	0.013	0.030	<0.0001	0.000
Acidity (%)	1.16 ^c^	1.38 ^a^	1.33 ^b^	1.33 ^b^	0.008	<0.0001	<0.0001	<0.0001
Nonfat solids (%)	8.43 ^b^	8.23 ^c^	8.55 ^b^	9.19 ^a^	0.042	<0.0001	<0.0001	<0.0001
Leucine (mg/100 g proteína)	3.23 ^c^	6.90 ^a^	5.53 ^b^	3.02 ^c^	0.092	<0.0001	<0.0001	<0.0001
Protein	4.48 ^a^	4.72 ^a^	4.13 ^b^	4.08 ^b^	0.078	<0.0001	0.049	0.306
Moisture	85.97 ^a^	84.81 ^b^	85.99 ^a^	83.95 ^b^	0.247	<0.0001	<0.0001	<0.0001

^a,b,c^ Means in the same row with different superscripts differ significantly between treatments. N, *Moringa oleifera* extract level; T, storage time; N × T, interaction of extract level and storage time; SE, standard error.

**Table 3:** Overall acceptance of yogurt from sheep fed with *Moringa oleifera* extracts.

	*Moringa oleifera* (mL)				SE	*p*-Value
	0	20	40	60		
Overall acceptance	2.99	2.95	3.94	3.88	0.990	0.137

SE = standard error.

## Data Availability

The data presented in this study are available on request from the corresponding author.
